# Targeting motility and chemotaxis as a strategy to combat bacterial pathogens

**DOI:** 10.1111/1751-7915.14306

**Published:** 2023-06-30

**Authors:** Miguel A. Matilla, Tino Krell

**Affiliations:** ^1^ Department of Biotechnology and Environmental Protection, Estación Experimental del Zaidín Consejo Superior de Investigaciones Científicas Granada Spain

## BACTERIAL PATHOGENS, ANTIMICROBIAL RESISTANCE AND GLOBAL FOOD SECURITY

Humanity keeps paying a high toll to bacterial pathogens. It has been estimated that 33 bacterial pathogens caused the death of 7.700.000 people in 2019 (GBD 2019 Antimicrobial Resistance Collaborators, 2022). In addition, plant pathogens cause an up to 40% reduction in the global crop production, leading to 4 million hunger‐related deaths annually (He & Creasey Krainer, 2020) and an economic loss of USD 220 billion per year (Savary et al., 2019). This toll may increase due to pathogen evolution and alterations of host‐pathogen interactions caused by climate change (Singh et al., 2023). Antibiotics and chemical pesticides are currently our main weapons to fight bacterial pathogens. However, their excessive and inappropriate use has fostered the alarming increase in the number of resistant strains, that underlines not only the necessity to develop new antibiotics and pesticides (Tacconelli et al., 2018), but also to identify alternative antimicrobial strategies (Krell & Matilla, 2022). Antibiotics either kill or slow down bacterial growth, resulting in an evolutionary pressure that favours the appearance of antibiotics‐resistant bacteria. As an alternative, anti‐infective therapy is based on targeting molecular mechanisms that lead to disease but that do not interfere with bacterial growth (Allen et al., 2014; Cegelski et al., 2008).

## THE CORE CHEMOTAXIS SYSTEM IS CONSERVED AMONG BACTERIA

Chemotaxis permits bacteria to move in compound gradients. Chemotaxis genes were detected in about half of the bacterial and archaeal genomes (Gumerov et al., 2020). There is almost no limit as to the type of signal that induces chemotaxis, including for example compounds that serve as N‐ and C‐sources, terminal electron acceptors, ions, neurotransmitters, quorum‐sensing molecules as well as human and plant hormones (Matilla et al., 2022). Through chemotactic movements, bacteria gain access to compounds of metabolic value and sample important information on their environment (Colin et al., 2021).

The core proteins of a chemotactic signalling cascade are conserved among bacteria (Wuichet & Zhulin, 2010). The central element is the ternary complex formed by chemoreceptors, the CheA histidine kinase and the CheW coupling protein. Signal binding to the chemoreceptor modulates CheA activity and the subsequent transphosphorylation of the CheY response regulator. CheY‐P binds to the flagellar motor to change its direction of rotation, which leads to a biasing of the swimming behaviour to navigate within the chemical gradient (Colin et al., 2021; Zhou et al., 2023). The sensitivity of the system is adjusted to the present signal concentration via the concerted action of the CheR methyltransferase and the CheB methylesterase. These six core proteins are present in almost all signalling cascades and their deletion or inactivation abolishes chemotaxis (Wuichet & Zhulin, 2010). In addition, some but not all chemotaxis signalling systems contain auxiliary proteins (Wuichet & Zhulin, 2010).

## CHEMOTAXIS AS A VIRULENCE DETERMINANT FOR BACTERIA WITH VERY DIFFERENT LIFESTYLES

Chemotaxis is essential for the virulence of many bacteria with very different lifestyles (Zhou et al., 2023). The majority of human pathogens defined by the World Health Organization (WHO) as priorities for the development of new antibiotics (Tacconelli et al., 2018) as well as 9 of the 10 most relevant phytopathogens (Mansfield et al., 2012) contain chemotaxis genes (Gumerov et al., 2020). The relevance of chemotaxis for pathogens of different lifestyle is illustrated here by several examples.

*Helicobacter pylori* is a human pathogen for which the role of chemotaxis in infection has been studied extensively. *H. pylori* infects the stomach causing gastritis, ulcers and cancer. Its chemotactic system senses a number of attractants and repellents that permit to localize preferred niches within the gastric mucosa. Next to its role in stomach colonization and early infection, chemotaxis was also found to modulate the host inflammatory response (Johnson & Ottemann, 2018). This is reflected by the fact that the deletion of each of its four chemoreceptors resulted in impaired virulence (Johnson & Ottemann, 2018).

*Borrelia burgdorferi* is the etiological agent of Lyme disease, an inflammatory infection resulting in cardiac, neurological and arthritic complications. The infectious cycle of *B. burgdorferi* is highly complex, with the pathogen cycling between the tick vector and the mammalian host. During its life cycle the pathogen not only has to migrate from the mid‐gut to the salivary glands within the tick to allow host transmission, but has also to navigate through the skin of the vertebrate host after the tick bite to reach different target tissues. Despite the small size of its genome, the pathogen has a sophisticated chemotaxis system which accounts for 5%–6% of its genome (Charon & Goldstein, 2002). A number of studies, exemplified by (Motaleb et al., 2015; Novak et al., 2016; Sultan et al., 2013, 2015), have shown that chemotaxis is essential for every stage of its infectious cycle.

*Pseudomonas aeruginosa* is an universal human pathogen that infects almost all tissues, but lung infections of cystic fibrosis patients are of particular clinical relevance. *P. aeruginosa* migrates rapidly to dying cells in scratch‐wounded human cystic fibrosis airway epithelial cells to initiate infection. However, migration and immobilization at wounds was abolished in a non‐chemotactic mutant and greatly reduced in a triple mutant in PctA, PctB and PctC (Schwarzer et al., 2016) – chemoreceptors that mediate chemotaxis to different amino acids (Gavira et al., 2020; Taguchi et al., 1997). The authors thus conclude that *P. aeruginosa* migration and binding to epithelial cells along wounds is strongly driven by amino acid chemotaxis (Schwarzer et al., 2016).

Many plant pathogens enter the host through stomata and wounds. There is an increasing body of data indicating that chemotaxis to compounds released by plant openings is essential for efficient entry and virulence (Matilla & Krell, 2018). For example, *Pseudomonas syringae* moves chemotactically towards open, but not closed stomata (Melotto et al., 2006). Another study showed that chemotaxis‐mediated entry through stomata was induced in the presence of light, which increased the production of photosynthetic products (Ranjbaran et al., 2020). The relevance of chemotaxis for entry into the host plant is also reflected by studies using different plant infection protocols. Leaf infection using spray inoculation (where bacteria need to localize openings) with non‐chemotactic and non‐motile *Ralstonia solanacearum* strains showed reduced virulence as compared to the wild type (Yao & Allen, 2006). However, when plants were infected by infusing these strains into the plant, no differences in the virulence between the wild type and the non‐chemotactic/non‐motile mutants were noted (Yao & Allen, 2006), underlining the relevance of chemotaxis in plant entry.

## IS INTERFERING WITH BACTERIAL MOTILITY A SUITABLE STRATEGY TO FIGHT PATHOGENS?

In the following section, we will briefly summarize studies demonstrating the use of different compounds to interfere with chemotaxis and/or motility. Due to space constrains, this is not intended to be a comprehensive review and only representative examples are cited. A number of different molecular mechanisms can be distinguished.

### Perturbing existing signal gradients reduces bacterial virulence

**FIGURE 1:**
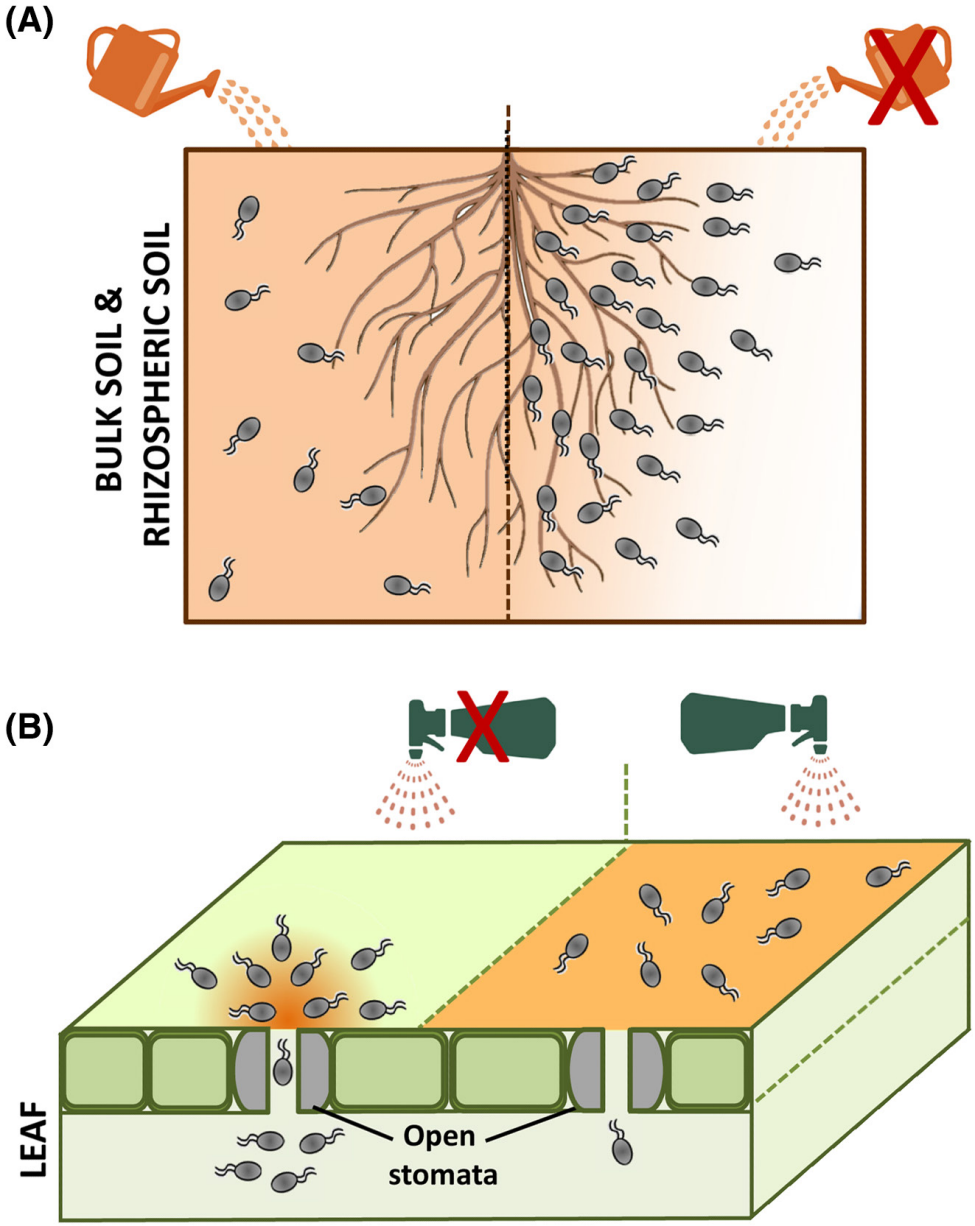
Perturbing host‐generated signal gradients reduces bacterial virulence. Schematic representation of the effect of perturbing chemoeffector gradients in the rhizosphere (A) and leaf surface (B) on root colonization and plant entry, respectively, of pathogenic bacteria. Based on studies reported by (Cerna‐Vargas et al., 2019; Tunchai et al., 2021).

*Ralstonia pseudosolanacearum* is a phytopathogen that causes bacterial wilt. Two of its chemoreceptors, McpA and McpM, were identified to mediate chemotaxis to most proteinogenic amino acids and L‐malate, respectively (Hida et al., 2015). Subsequent root infection assays revealed a large reduction in virulence for the mutant in *mcpM*, but not for the *mcpA* mutant, suggesting that chemotaxis to L‐malate, but not to amino acids, is required for chemotaxis towards the root and subsequent plant infection (Hida et al., 2015). The authors thus asked whether saturating malate concentrations, masking malate gradients in the rhizosphere, might reduce the virulence of the wild type strain (Figure 1A). This hypothesis was verified by infection assays with bacteria containing 1 mM malate, that resulted in a reduction by 40%–50% of the severity of the disease caused by this phytopathogen (Tunchai et al., 2021).

Similar observations were made with another plant pathogen, *P. syringae* (Cerna‐Vargas et al., 2019). The chemoreceptor PsPto‐PscA bound specifically, and with high affinity, L‐Asp, L‐Glu and D‐Asp. In analogy to the above study, the PsPto*‐pscA* mutant showed a reduced virulence phenotype as well as reduced entry into leafs. Plant leaf infection assays were thus repeated in the presence of saturating concentrations of these three ligands (Figure 1B), revealing a significant drop in bacterial virulence and plant entry in the presence of D‐Asp, but not in the presence of the remaining two amino acids (Cerna‐Vargas et al., 2019). In contrast to L‐Asp and L‐Glu, D‐Glu is not metabolized by *P. syringae*, which was hypothesized to account for the differential effect of the three ligands in virulence (Cerna‐Vargas et al., 2019). The term “chemotactic disruption” was primed and this strategy, based on masking of natural compound gradients (Figure 1), was proposed to be an effective alternative strategy to fight plant pathogens (Cerna‐Vargas et al., 2019; Tunchai et al., 2021).

An analogous strategy has also been used to combat a human pathogen. *H. pylori* is repelled by the very low pH in the stomach, that is generated by acid secretion from gastric glands, and moves chemotactically to the protective mucus layer covering the stomach surface. Two acid‐sensing chemoreceptors, TlpA and TlpD, play a key role in mediating this chemotactic response. Thus, a *tlpAD* double mutant, that was unable to sense acidic pH, is deficient in its ability to colonize the stomach of mice (Huang et al., 2017). Treatment with omeprazole, a proton pump inhibitor that raises the gastric pH, not only restored partially the stomach‐colonizing capacity of the mutant strain, but also altered the localization of *H. pylori* in the stomach, allowing it to move deeper into the gastric glands (Huang et al., 2017). It has been proposed that this disorientation of *H. pylori* is responsible for the synergistic effect observed during the combined treatment of omeprazole with different antibacterial drugs (Zhou et al., 2023).

### Inhibition of swimming motility by compounds that bind to the flagellar motor

**FIGURE 2:**
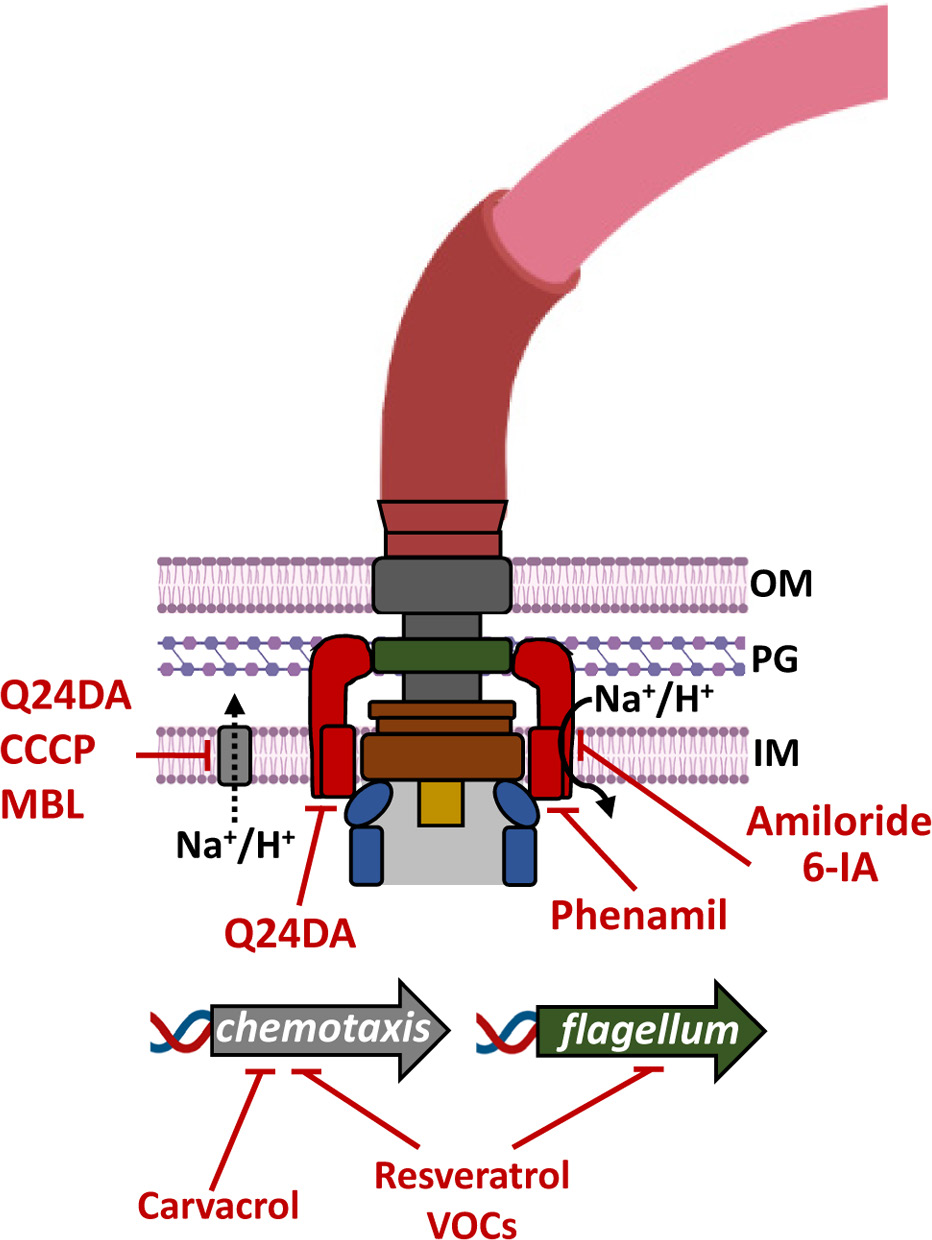
Targeting chemotaxis and motility as an anti‐infective therapy strategy. Schematic representation of a bacterial flagellum (based on that of *Salmonella enterica* (Minamino et al., 2019)). Several compounds that interfere with motility and chemotaxis by binding to the flagellar motor, perturbing Na^+^ and H^+^ gradients and reducing the expression of genes/proteins involved in chemotaxis and flagellar synthesis/functioning are shown. 6‐IA, 6‐iodoamiloride; CCCP, Carbonyl cyanide *m*‐chlorophenylhydrazone; IM, inner membrane; MBL, mannose‐binding lectin; OM, Outer membrane; PG, peptidoglycan; Q24DA, quinazoline‐2,4‐diamino analog; VOCs, volatile organic compounds.

The flagellum is a highly complex assembly of proteins encoded by about 40 different genes (Manson, 2022). Over the years, a number of compounds were identified that bind to the flagellar motor inhibiting its activity (Figure 2). Initially, the diuretic drug amiloride, a pyrazinoylguanidine derivative, was reported to inhibit rotation of the sodium‐powered flagellar motor by blocking the translocation of Na^+^ ions through the channel in a competitive and rapidly reversible manner. This compound completely inhibited motility, but showed almost no effect on the membrane potential, the intracellular pH and the ATP content of the cell (Sugiyama et al., 1988). Like amiloride, its derivative 6‐iodoamiloride (6‐IA) reversibly inhibited Na^+^ driven motors. However, when 6‐IA‐treated cells are irradiated with UV light, the inhibition is irreversible. Data suggest that photoactivated 6‐IA binds specifically and covalently at or around the Na^+^ binding site to inhibit motor function (Muramoto et al., 1994). The amiloride analogue phenamil has then been identified as an inhibitor of Na^+^ powered motors (Atsumi et al., 1990). Phenamil stops flagellar rotation by binding to the cytoplasmic face of the Na^+^ channel components of the stator complex (Kojima et al., 1999). Similarly, a quinazoline‐2,4‐diamino analogue (Q24DA) also inhibited swimming motility of *Vibrio cholerae* by binding to components of the stator complex (Wang et al., 2013). In a more recent study, two phenamil analogues were developed that suppressed motility by inhibiting Na^+^ and H^+^ driven stators in pathogenic and nonpathogenic strains (Islam et al., 2021). There was an increase in the efficiency of these phenamil analogues with respect to amiloride. Whereas 500 μM amiloride were required for an inhibition of the motor (Sugiyama et al., 1988), complete suppression of motility was observed at 50 μM phenamil (Atsumi et al., 1990) and the recently developed phenamil analogues almost abolished motility at 10 μM (Islam et al., 2021).

### Inhibition of the motor be perturbing Na^+^ and H^+^ gradients

Motors are driven by Na^+^ or H^+^ gradients and the interference with the generation of these gradients is another mechanism to reduce motility (Figure 2). For example, an ~8000 compound library was used to screen for defects in *Vibrio cholerae* motility, resulting in the identification of another Q24DA as an inhibitor that acted specifically on Na^+^‐driven motors. The corresponding IC_50_ value was in the lower micromolar range (Rasmussen et al., 2011). Further experiments showed that this inhibition is not due to motor binding, but to an interference with Na^+^ bioenergetics (Rasmussen et al., 2011). Carbonyl cyanide *m*‐chlorophenylhydrazone (CCCP) is a potent H^+^ pump inhibitor. A number of studies show that CCCP inhibited chemotaxis, namely that of *H. pylori* (Yoshiyama et al., 1998), *Escherichia coli* (Schauer et al., 2018), or *Pelotomaculum thermopropionicum* (Kosaka et al., 2019), indicating an inhibition of the flagellar motor by interfering with the generation of H^+^ gradients. The mannose‐binding lectin (MBL) is present in the serum of warm‐blooded animals and plays a key role in the innate immune response (Kalia et al., 2021). It was shown that physiological concentrations of MBL reduced *Salmonella enterica* chemotaxis. This was due to a reduction in swimming speed and flagellar rotation caused by a perturbation of the H^+^ gradient required for motor function (Xu et al., 2016). MBL also acted on transmembrane chemoreceptors by altering their activity and causing an increase in flagellar reversal frequency. The authors propose that the reduction in motility and chemotaxis of a pathogen by an innate immune protein represents a defence mechanism (Xu et al., 2016).

### Inhibition of swimming by reducing chemotaxis protein and/or transcript levels

There are numerous studies of compounds that reduce chemotaxis and swimming behaviour by reducing levels of chemotaxis proteins (Figure 2). For example, the plant‐derived polyphenol resveratrol reduced swimming motility of avian pathogenic *E. coli*, which was caused by lowering the cellular abundance of chemoreceptors, chemotactic signalling proteins, flagellin and motor proteins (Ruan et al., 2021). Volatile organic compounds (VOCs) produced by different plant bacterial isolates of the *Bacillus* genus strongly reduced swimming, swarming and twitching motility, as well as chemotaxis towards root exudates of the phytopathogen *R. solanacearum* (Tahir et al., 2017). These VOCs caused important reductions in the transcript levels of several motility and chemotaxis genes (Tahir et al., 2017). As mentioned above, chemotactic motility of *R. solanacearum* is essential for efficient plant infection (Yao & Allen, 2006). Similarly, sub‐inhibitory concentrations of the phenolic monoterpenoid carvacrol, an active constituent of oregano oil, reduced *Campylobacter jejuni* motility, which is likely due to the reduction in the cellular levels of proteins required for chemotaxis such as chemoreceptors (Wagle et al., 2020). Another example of the potential of natural compounds to inhibit swimming motility is the observation that the co‐culture of *Vibrio parahaemolyticus* with the algae *Ulva fasciata* inhibited swimming and twitching. This reduction was attributed to the reduced transcript levels of multiple chemotaxis signalling and flagellar motor genes (Qiao et al., 2022). The active compound has not been identified.

Frequently, the economic cost is a major obstacle for the development of novel antimicrobial agents. Another study showed that a derivative of an agricultural waste product, pomegranate peel juice, reduced swimming and swarming motility of *Salmonella enterica* sv. Typhimurium. Treatment with pomegranate peel extracts reduced about 5‐fold the number of flagellated cells and flagella per cell, a phenotype that was associated with a significant reduction in the expression of flagellar genes (Mahadwar et al., 2015). Further work is required to identify the active components in these plant extracts.

### Inhibition of biofilm formation by inhibiting swimming motility

Biofilm formation is closely related to bacterial virulence and contributes to the bacterial resistance against antimicrobial agents (Ciofu et al., 2022). Bacterial motility is often an essential requisite for efficient biofilm formation (Colin et al., 2021) and several studies relate a reduction in biofilm formation to decreases in motility. For example, *Listeria monocytogenes* forms biofilms at many different surfaces and at all stages of the food‐processing chain (Cherifi et al., 2017). Structurally very diverse compounds like cell‐free supernatants of coagulase‐negative staphylococci, ZnCl, EDTA or the plant‐derived alkaloid tomatidine were found to inhibit biofilm formation (Doghri et al., 2021). To identify the corresponding molecular mechanism, the authors demonstrated that all compounds caused a significant reduction in swimming motility, underlining the importance of motility in biofilm formation (Doghri et al., 2021). Another study revealed that treatment with agaric acid, a natural fungal fatty acid, dramatically inhibited the biofilm‐forming capacities of *S. enterica* sv. Typhimurium – a phenotype that correlated with a reduction in the expression of flagellar genes and with the inhibition of swimming motility in the presence of the compound (Lories et al., 2020). In a similar manner, a small cationic peptide was found to inhibit *P. aeruginosa* biofilm formation by reducing swimming and swarming motilities (de la Fuente‐Núñez et al., 2012).

## IS THE INTERFERENCE WITH BACTERIAL MOTILITY A SUITABLE ALTERNATIVE STRATEGY TO FIGHT PATHOGENS? ARGUMENTS IN FAVOUR AND AGAINST

A variety of targets are currently explored to combat pathogens. Future medicines may be cocktails of different compounds with different antimicrobial mechanisms. The inclusion of agents that reduce bacterial motility may thus be an option. What are thus the arguments in favour and which facts may advocate against the use of motility inhibitors for anti‐infective therapy?

### Arguments in favour

1. The chemotactic mechanism is highly complex providing many target proteins for interference, such as chemoreceptors, chemosensory signalling proteins, flagellar motor proteins and transcriptional regulators. The proof of concept of inhibiting chemotaxis by interfering at different levels has been obtained.
2. The molecular mechanism of chemotaxis is highly conserved in bacteria. Core proteins of a chemosensory signalling cascade (e.g. chemoreceptors, CheAWYBR) and the flagellar motor are conserved, enhancing the possibilities for the development of broadly active agents.
3. IC_50_ values of motility/chemotaxis‐inhibiting compounds of around or below 10 μM have been obtained. Compounds that perform inhibition at such concentrations are generally considered hits, that then can be used to develop analogues with lower IC_50_ values.
4. In some important pathogens like *P. aeruginosa*, chemotaxis and twitching motility are mediated by signalling cascades composed of homologous proteins. Interfering with chemotactic signalling may thus also impair twitching – a form of motility that in some bacteria has been shown to be important for virulence.
5. Many of the compounds that inhibit motility do not inhibit growth. The lacking or weak impact of many compounds on bacterial growth will thus not favour the generation of resistant mutants.

### Arguments against

1. Chemotaxis genes are found in only about half of the bacteria and some important pathogens are non‐chemotactic. The targets for inhibition are thus not as omnipresent as, for example, the ribosome, which may hinder the development of broadly active compounds.
2. Whereas for some pathogens the deletion of central chemotaxis genes abolished virulence, in a number of other pathogens, suppression of chemotaxis reduced but not abolished bacterial virulence.
3. Because motility inhibitors do not kill or inhibit the growth of the pathogen, their use will not result in eradication of the target microorganism.

## FINAL REMARKS

Combating pathogens with a set of drugs, combining different inhibitory targets and mechanisms of action, rather than with an individual drug, is a promising strategy to combat pathogens (Bari et al., 2023; Sanz‐García et al., 2023). In addition, targeting microbial virulence mechanisms instead of survival, is likely to reduce the emergence of antimicrobial resistant strains (Allen et al., 2014). Both, motility (Josenhans & Suerbaum, 2002) and chemotaxis (Matilla & Krell, 2018) are required for the virulence of numerous bacterial pathogens at different stages of the infective process. Pondering the different advantages and disadvantages as well as the current experimental evidence indicates that the interference with bacterial motility and chemotaxis is a reasonable strategy to fight pathogens. Furthermore, the combined use of anti‐virulence compounds and classical antimicrobials may enhance the activity and lifespan of existing antimicrobials (Miethke et al., 2021). This notion also applies to compounds that interfere with motility, as shown by the motility inhibitor resveratrol that increased the antimicrobial activity of certain classes of antibiotics (Vestergaard & Ingmer, 2019). The chemotactic machinery is highly complex, providing a large number of targets of which only some have been explored. Therefore, further research in this field is needed to explore these targets and to translate the gathered knowledge into an efficient therapeutic approach of clinical and agricultural relevance.

## AUTHOR CONTRIBUTIONS

**Miguel A. Matilla:** Conceptualization (equal); funding acquisition (equal); writing – original draft (equal); writing – review and editing (equal). **Tino Krell:** Conceptualization (equal); funding acquisition (equal); writing – original draft (equal); writing – review and editing (equal).

## CONFLICT OF INTEREST STATEMENT

The authors do not declare a conflict of interest.
